# Tantalum as Trabecular Metal for Endosseous Implantable Applications

**DOI:** 10.3390/biomimetics8010049

**Published:** 2023-01-23

**Authors:** Filippo Carraro, Andrea Bagno

**Affiliations:** Department of Industrial Engineering, University of Padova, 35131 Padova, Italy

**Keywords:** tantalum, trabecular metal, porous metal, endosseous devices, osteointegration

## Abstract

During the last 20 years, tantalum has known ever wider applications for the production of endosseous implantable devices in the orthopedic and dental fields. Its excellent performances are due to its capacity to stimulate new bone formation, thus improving implant integration and stable fixation. Tantalum’s mechanical features can be mainly adjusted by controlling its porosity thanks to a number of versatile fabrication techniques, which allow obtaining an elastic modulus similar to that of bone tissue, thus limiting the stress-shielding effect. The present paper aims at reviewing the characteristics of tantalum as a solid and porous (trabecular) metal, with specific regard to biocompatibility and bioactivity. Principal fabrication methods and major applications are described. Moreover, the osteogenic features of porous tantalum are presented to testify its regenerative potential. It can be concluded that tantalum, especially as a porous metal, clearly possesses many advantageous characteristics for endosseous applications but it presently lacks the consolidated clinical experience of other metals such as titanium.

## 1. Introduction

The worldwide increase in the elderly population is testament to the general improvement of living conditions but necessitates a parallel increase in healthcare actions. With specific regard to the orthopedic field, the Italian Arthroplasty Registry (RIAP) evidences an average growth of orthopedic surgeries by 7.2% from 2018 to 2019, in detail: +5.7% for the hip, +8.8% for the knee, and +20.3% for the shoulder [1]. As documented in [2,3], the worldwide market of orthopedic devices (both accessories and surgical apparatuses) was valued at 40.9 billion USD in 2021, and it is expected to reach approximately 43.1 billion USD by 2024. This is due to multiple reasons: the growing aging population of course, but also the incidence of orthopedic disorders (e.g., degenerative bone disease) caused primarily by sedentary lifestyle and obesity, and the rising number of road accidents. Consequently, manufacturers are investing significantly in the development of more efficient devices with the aim of reducing the costs and introducing technological innovations at the same time.

In this context, the search for innovative materials becomes a crucial item to overcome health-related problems, thus increasing patients’ quality of life [4]. Any material intended for use in permanent contact with bone has to “respect” its complex physiology, which ultimately depends on the interconnected roles of osteoblasts and osteoclasts [5]. While osteoblasts are mainly responsible for bone tissue deposition, osteoclasts degrade and resorb mature bone. The fine balancing of these opposite activities allows regulating the so-called “bone remodeling” [6]; this implies the removal of mineralized bone and the formation of newly deposited bone matrix (where mature osteoblasts are embedded within the lacunae and eventually differentiate into osteocytes). Bone remodeling allows adjusting the bone architecture in response to variable mechanical stimuli; it also permits repairing micro-damages to prevent their dangerous accumulation. Lastly, bone remodeling is fundamental to maintain calcemic levels by releasing calcium ions from the bone matrix during degradation, and accumulate them during mineralization.

Being able to convert a mechanical information (load) into a biological activity (remodeling), bone cells are capable of mechanotransduction [7]. If the bone tissue is not properly solicited, e.g., because of the presence of a prosthetic device altering load distribution, the correct remodeling is hampered. Consequently, atrophic bone is produced where the tissue is no more physiologically loaded, and denser bone grows in the area exposed to stresses higher than physiological [8]. This phenomenon is called the “stress-shielding effect”. The capacity of the healthy bone to adapt to variable loading conditions was discovered and described in the 19^th^ century by the well-known Wolff’s law [9]; indeed, the exact mechanism of bone adaptation to load has not yet been completely understood [10].

Endosseous devices, such as those commonly used in orthopedics, have to assure adequate mechanical features and biocompatibility; moreover, they have to promote biological fixation and possibly prevent the stress-shielding effect. On the one hand, materials traditionally used in orthopedics (e.g., stainless steel, cobalt–chromium, and titanium alloys) provide excellent structural support with very good clinical outcomes; on the other hand, their high stiffness and low porosity represent major unsolved limitations [11]. With regard to orthopedic applications, it is worth mentioning the use of nitinol (e.g., nickel and titanium alloy characterized by the shape memory effect [12]) for the production of arthrodesis implants experimentally evaluated both on a polyurethane foam model and on a cadaveric model [13,14].

Materials other than metals can be exploited for endosseous devices, as well as for blood-contacting applications: for instance, silicon carbide (SiC), due to its inertness, hardness, stiffness, tribological features, and hemocompatibility, has been considered as an alternative to traditional metallic materials such as CoCrMo and Ti6Al4V alloys [15].

Over time, other metallic materials have been proposed. Due to its high resistance to corrosion and excellent biocompatibility, the potential of tantalum (Ta) has emerged for several biomedical applications since the 1940s, particularly surgical suture threads, bone fixation parts, bone implants, vascular stent coatings, and medical imaging contrast agent [16]. Moreover, tantalum exhibits advantageous mechanical ductility, combined with the potential for osteoconductivity, osteoinductivity, and angioinductivity. While Ta presents a relatively high stiffness (185 GPa) in the solid form, when it is produced as a porous material, its elastic modulus decreases to 3 GPa, which is similar to that of human bone (from 0.4 GPa of trabecular bone to 17.9 GPa of cortical bone) [17,18].

By definition, porous metals (or trabecular metals) present a cellular structure characterized by a three-dimensional network of interconnected pores; they are of particular interest for orthopedic applications [19]. Porous metals exhibit lower but sufficient stiffness and strength compared to solid metals; therefore, they are mainly used for load-bearing and structural purposes. Indeed, any specific application depends on three parameters: composition, macroscopic shape, and pore structure [20]. The composition determines the capacity of the material to suit physical and chemical requirements. The macroscopic shape is critical for the integration/combination with other materials. The pore structure mainly affects the mechanical stability and, in the special case of orthopedic implants, the accommodation of bone cells. Thus, in addition to other advantageous features, porous Ta is intrinsically able to support attachment, proliferation, differentiation, and mineralization of osteoblasts, thus promoting osteogenesis and osteointegration [21]. 

The present paper aims at reviewing the peculiar properties and features of tantalum as a porous metal and its applications for the production of orthopedic/dental devices; particular attention is focused on biocompatibility and bioactivity issues. 

### Methodological Approach and Scope

A literature survey through the MEDLINE database was performed between August 2022 and December 2022. No date restrictions were specified. The following keywords were applied: “tantalum”, “porous tantalum”, “porous tantalum trabecular metal”, and “porous tantalum osteogenesis”. MEDLINE returned 4623 items searching for “tantalum”; this number decreased to 525 with “porous tantalum”, to 118 with “porous tantalum trabecular metal”, and to 47 with “porous tantalum osteogenesis”. Google Scholar was also reviewed to extend the number of possible citations. Papers were then selected for relevance, and the article’s references were also examined.

## 2. Tantalum and Its Properties

Tantalus was a Greek mythological figure who was condemned to a famous punishment; he had to stand in a water pool under a tree, but he was not allowed to drink the water or to eat the fruits. The myth illustrates the refractory property of tantalum that is highly unreactive in almost all acids, with the exception of hydrofluoric acid and acids containing fluoride and sulfur trioxide [22].

Tantalum is a chemical element with atomic number 73, and with a molecular mass equal to 180.05, represented by the symbol Ta [18]. It was discovered by Anders Ekeberg in 1802 and isolated by Berzelius Jöns in 1820. It is a transition metal present in the Earth’s crust (1–2 ppm) [23]; it is a rather rare metal, found in the minerals tantalite and euxenite. Tantalum is malleable at room temperature and has a bright blue-gray color. Being a refractory metal, it has a high melting point (3017 °C) and can be used as a thermal and electrical conductor. Tantalum quickly reacts with oxygen to form oxides, which exist in two forms: Ta_2_O_5_ and TaO_2_. When tantalum is exposed to the air or is industrially processed, it spontaneously tends to form a layer of Ta_2_O_5_ (passivation), which is not conductive, allowing the material to be highly resistant to acids and bases. For this reason, tantalum is not very soluble for any pH and potential value [18].

Tantalum and its alloys possess relevant physicochemical properties, which make them suitable for the production of prosthetic implants and for many other biomedical applications. In particular, the following aspects are considered herein: mechanical properties, corrosion resistance, radiopacity and MRI compatibility, surface properties, hemocompatibility, and osseointegration potential.

### 2.1. Mechanical Properties

The mechanical properties of tantalum are summarized in Table 1. Tantalum exhibits a remarkable elastic modulus (185 GPa), even higher than commercially pure titanium and close to that of 316 L stainless steel and cobalt–chromium (CoCr) alloy. On the other hand, yield strength and tensile strength are much lower than those of the abovementioned materials.

### 2.2. Corrosion Resistance

When any metal is implanted into the human body, it has to face an aggressive environment that usually triggers corrosion. Corrosion is the sum of redox reactions that take place in the presence of oxygen in an electrolytic solution, which ultimately result in the release of metal ions and in the degradation of the metallic device. Metal ions can cause toxic effects to the surrounding biological tissues, even systemically. The degradation of the metallic device is accompanied by a progressive loss of physical, chemical, and mechanical features. Therefore, corrosion resistance is of paramount importance for all metals intended for the production of implantable devices.

Passivation is a mechanism to increase the corrosion resistance; it consists of the formation of a very compact oxide layer that firmly adheres to the metallic surface. The oxide layer can protect the underlying metal from the direct contact with the biological fluids; moreover, due to its high electrical resistance, it also avoids the transfer of electrons that sustain the redox reactions. As the removal or degradation of the oxide protective layer is often very difficult, corrosion in passivated metals is effectively stopped. 

The excellent corrosion resistance of Ta is due to the formation of a stable and dense layer of tantalum oxide (Ta_2_O_5_), about 2–3 nm thick, which prevents the release of metal ions. Furthermore, a TaO_2_ film is also present between the overlying Ta_2_O_5_ layer and the underlying metal. Several studies highlight the excellent corrosion resistance of Ta and its alloys in acidic and basic environments, particularly in the presence of HCl, H_3_PO_4_, and NaOH [24,25,26,27]. All these studies also demonstrated that the corrosion rate increases with temperature and acid concentration in solution, but adding Ta alloyed with other elements improves the corrosion resistance.

In the study published by Silva et al. [28], the characteristics of the surface oxide layer of Ta in a 0.15 M NaCl solution (simulated body fluid, SBF) were investigated. Discs of pure Ta (15 mm diameter, 5 mm thickness) were immersed in the solution together with the electrodes. After 1 h, the anodic polarization curve showed an initial current increase with a maximum at 1.65 V followed by a decrease; the current growth corresponds to the local dissolution of Ta associated with the degradation of the passivating film, while the decrease is due to the repair of the film thanks to repassivation. 

### 2.3. MRI Compatibility

Ta is characterized by high radiopacity thanks to its atomic number and density. Therefore, implantable prosthetic structures made of this metal are easily monitored with fluoroscopy. For example, this imaging technique allows ensuring proper stent release during angioplasty procedures. Table 2 summarizes the atomic numbers and density values of some metals frequently used as biomaterials.

The magnetic resonance imaging (MRI) procedure cannot be performed when ferromagnetic materials are present; indeed, the MRI acts like a strong magnet that attracts these kinds of materials very violently. This is a clear limitation for the application of diagnostic imaging techniques to form pictures from metallic implantable devices. Ta is not affected by this drawback being a nonferromagnetic metal. However, most of the power transmitted during MRI is converted into heat inside the patient’s body; the metallic device can be heated up, depending on the size and geometry of the implant. It is important to calculate the amount of heat generated during MRI and to predict any possible adverse effect [22]. Two studies [29,30] calculated the temperature variation inside the human body in order to check Ta compatibility during MRI examinations of different stents. A maximum temperature rise of 0.3 °C was observed under typical MRI conditions (1.5 T), thus preventing any risk for the biological structures. Moreover, when it is used as a marker in ophthalmology, Ta is still compatible with MRI even for higher magnetic field values (7 T).

### 2.4. Surface Properties

The biological response to any implantable material is closely related to its surface properties, including surface energy, morphology, charge, and chemical composition. Surface energy determines the amount of attractive or repulsive forces that the surface can exert on another material. In general, metals have a high surface energy (500–5000 mN/m) due to the presence of the metallic bond. The amount of surface energy controls protein adsorption and the subsequent phases of cell adhesion and growth. The study by M. M. Gentleman and E. Gentleman [31] stated that materials with high surface energy promote cell adhesion and growth, while materials with low surface energy (e.g., some polymers) do not.

Tantalum’s surface energy is 100.59 ergs/cm^2^; this high value is supposed to be a determining factor for promoting the adhesion and growth of osteoblasts and for the osseointegration potential of this metal. Some manufacturing processes can decrease the surface energy for the use of tantalum in contact with blood (e.g., chemical vapor deposition, diffusion coating, nanotube formation, and fluorination). 

### 2.5. Hemocompatibility

Surface chemical composition significantly affects the surface energy of any material. In the case of tantalum, after passivation, an oxide layer composed of Ta^5+^ and O^2−^ ions covers the surface. When these ions attract water molecules, the surface becomes hydrophilic, and this implies a high surface energy. A smooth oxide layer is enough to prevent thrombotic effects when tantalum is in contact with blood, while a very porous structure is used for the surface of orthopedic implants to promote osseointegration.

The isoelectric point of tantalum oxide is at 2.7–3.0; thus, its surface is negatively charged at physiological pH (7.4). Platelets, which are negatively charged at physiological pH, are repelled from the surface and this improves the hemocompatibility of the metal. Moreover, tantalum oxide can prevent the electronic transfer to fibrinogen [32], which is the precursor of fibrin, responsible for stabilizing the platelet plug forming the blood clot. This is the reason why the tantalum surface is deemed non-thrombogenic.

### 2.6. Osseointegration Potential

The term “osseointegration” refers to the intimate connection between any endosseous prosthetic implant and the surrounding bone tissue; it implies both the anatomical congruence of the device with respect to the biological environment and the ability to bear physiological loads. With regard to trabecular scaffolds, effective long-term functionality of the implant can be guaranteed by bone ingrowth within the porous structure and not only on the outer surface. The particular structure of trabecular Ta not only facilitates the adhesion and proliferation of osteoblasts, but it also favors the supply of nutrients and oxygen (as well as the removal of catabolites and CO_2_) that are necessary for new bone tissue formation. This latter begins with the differentiation of pre-osteoblasts and ends with bone mineralization; these processes involve a large number of genes and proteins related to osteogenesis [33].

Bone tissue regeneration is a complex mechanism involving the activation or inhibition of multiple signaling pathways. Interestingly, it has been acknowledged that Ta itself is associated with a large chain of signaling events typical of osteogenesis. Ta contributes to osteogenesis through the regulation of different cellular signaling pathways: the Wnt/β-catenin signaling pathway [34,35], transforming growth factor-beta (TGF-β) and bone morphogenetic proteins (BMPs) signaling pathway [36,37], mitogen-activated protein kinases (MAPKs) signaling pathway [38], and integrin signaling pathway [39].

The superior osteoinductivity of Ta to that of titanium received thorough investigations; for instance, the effects of Ta and Ti surfaces on osteogenesis using rat bone mesenchymal stromal cells (rBMSCs) were assessed by Lu et al. [37]. These authors also elucidated the molecular mechanisms regulating metal–cell interactions, which are basically mediated by the integrin α5β1/ERK1/2 pathway.

Hu Qian et al. recently reviewed all the mechanisms induced by Ta in osteogenesis [40]. This paper also pinpointed that many studies elucidated the role of Ta but with various limitations. Firstly, some investigations were just preliminary, while some mechanisms were not described in detail. Secondly, the involvement of Ta in promoting osteogenesis via other pathways associated with bone remodeling has not yet been fully proven.

## 3. Trabecular Tantalum

Two trabecular metals are commonly used for endosseous prosthetic implants: Ta and Ti (and their alloys). Since trabecular Ti shows some limitations, such as low porosity, low coefficient of friction, and an elastic modulus different from that of bone, trabecular Ta is usually preferred. It is characterized by a three-dimensional structure with high porosity (open cell); cells are repeated in a dodecahedral shape similarly to spongy bone. It is obtained via vapor deposition/infiltration of commercially pure Ta onto a vitreous carbon scaffolding. During manufacturing, the glassy carbon bearing structure can be modified to obtain a variety of configurations for a variety of orthopedic applications [11]. Trabecular Ta possesses a high porosity (75–80%), a high coefficient of friction (−1), and an elastic modulus much lower than that of compact Ta (~185 GPa) and more similar to that of bone (Table 3).

It is possible to control, to a certain extent, the mechanical properties of trabecular Ta by modifying its structure and changing the manufacturing technique. Indeed, different structural morphologies differ with respect to compressive strength. The elastic modulus can be altered depending on the fabrication process and on the porosity; for example, scaffolds with cubic pores exhibit higher modulus than scaffolds with diagonal pores. Furthermore, the elastic modulus increases as the porosity decreases and the diameter of the interconnection points increases. Porosity also determines the bending strength and the tensile strength, which decrease when porosity increases. In the work by Fan et al. [41], four types of Ta and Ti scaffolds with four pore diameters (1000–700 μm; 700–1000 μm; 500–800 μm; 800–500 μm) were produced by means of a selective laser melting technique; their responses to load were compared under uniaxial compression tests. Ta-based scaffolds revealed a mechanical behavior more similar to Ti-based scaffolds to that of pig bone.

### 3.1. Manufacturing Techniques

Tantalum has good mechanical properties and excellent biocompatibility, is resistant to corrosion, and can play a beneficial role in osteogenesis. All these advantages have been counterbalanced by its difficult manufacturing in the solid (compact) state due to the high melting point and high affinity for oxygen. Only from the early 1990s, thanks to the development of the porous tantalum trabecular-structured metal (PTTM), did this material begin to be used for several prosthetic applications [21]. Therefore, specific manufacturing techniques have been introduced and optimized to address each clinical purpose [20].

#### 3.1.1. Chemical Vapor Deposition (CVD)

Chemical vapor deposition (CVD) allows the deposition on a solid support of a molecular precursor, which is supplied in gaseous form and decomposes on the substrate surface. CVD is one of the most important techniques for coating several kinds of materials, and it is commonly used to produce Ta scaffolds in the clinical setting. CVD brings together a set of techniques aimed at depositing thin protective films on a surface; multiple methods are available depending on the process parameters chosen (i.e., pressure, temperature, and type of deposition) [42].

The first step is the production of a low-density vitreous carbon skeleton with a porosity of 98%; it is obtained by pyrolysis of a polymeric foam. The carbon skeleton shows a matrix where a pattern of dodecahedral interconnected pores is repeated. Since the carbon structure can be built with different shapes and sizes, several geometries for as many clinical applications can be produced. Thereafter, TaCl_5_ (Ta precursor) reacts with H_2_ at high temperature; Ta then deposits over the carbon structure to get a porous scaffold (99% Ta and 1% glassy carbon by weight). The thickness of the Ta coating varies from 40 to 60 μm, and this parameter can alter the porosity and the mechanical properties of the implant. Generally, the average pore size for orthopedic applications is between 400 and 600 μm and the porosity ranges from 75% to 85% [19]. Different materials other than carbon can be used as a substrate for Ta deposition; in [43], porous Ta scaffolds were produced coating porous silicon carbide (SiC) substrates through CVD at low temperature. The substrate is ultrasonically washed with an acidic solution (HF and HNO_3_), and then dried under nitrogen. TaCl_5_ (99.95% pure) is preheated to 223 °C in a stainless sublimator; H_2_ (99.999%) is used as both a carrier and a reducing agent, while argon (99.998%) is fluxed as a protective gas. TaCl_5_ vapor is carried by the hydrogen flow through the heated TaCl_5_ powders. The reaction between TaCl_5_ and H_2_ occurs at 1000 °C under a pressure of 2.5 Torr. The deposition process takes approximately 30 min; then, coated samples are ultrasonically cleaned with methanol, acetone, and distilled water.

The characteristics of the pores and, consequently, the mechanical properties of the porous structure can be partly tailored by controlling the thickness of the Ta layer deposited onto the substrate.

#### 3.1.2. Powder Metallurgy

**Figure 1:**
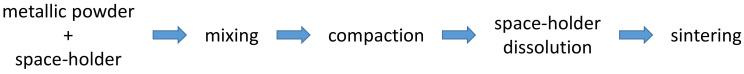
The main steps of the space-holder fabrication technique.

Powder metallurgy (PM), also known as the “space-holder method”, is a fabrication technique for porous Ta scaffolds associated with low fabrication costs. It consists of a sequence of steps that lead to the compaction and transformation of a metallic powder into a sintered material. This technique consists of five main steps (Figure 1): obtaining the powders, mixing them with the space-holder, compacting the powders, dissolving the space holder particles, and sintering. The Ta powders and the space-holding particles (particles that are later dissolved to generate pores) are mixed, and the mixture is compacted under appropriate pressure (350–450 MPa). The compact compound (green compact) is immersed in distilled water (at 60 °C) to dissolve the space-holding particles and obtain a porous structure Eventually, the porous structure is dried in an oven for 2 h and sintered at 1300–2000 °C under vacuum to obtain the final porous scaffold. The space-holding particles must possess the following features: low cost, fast dissolution in (hot) water, low melting point, non-cytotoxicity and low corrosive effects toward the metal during dissolution. The most used substances are starch, urea, sodium chloride, sucrose, and ammonium bicarbonate.

The effectiveness of porous Ta scaffolds obtained via the PM technique in orthopedic applications was illustrated in [44,45], where NaCl was used as space-holder. In detail, the authors analyzed the effect of NaCl particles on the mechanical properties of Ta scaffolds by varying their content (0, 30, 50, and 70) and granulometry (80–150 μm). Interestingly, when 30% or 50% of NaCl by volume was added, the mechanical properties of the scaffolds matched those of spongy bone (yield strength 6.6–36.2 MPa; elastic modulus 0.13–1.08 GPa). Thus, the porosity and mechanical properties of Ta scaffolds were determined by the size of the space-holding particles and their content in the initial powder mixture. Indeed, Ta scaffolds produced by PM have pore interconnectivity lower than those fabricated by CVD: the PM technique tends to generate a high number of closed pores. It is worth mentioning that the presence of closed pores markedly affects the osteoconductivity of the scaffold.

Another study [46] investigated the effect of porosity, space-holder particle size (NaCl), and compaction pressure on the morphology and mechanical properties of the porous Ta produced by means of PM. Ta powders and NaCl particles were weighted to obtain a porosity value around 60–80% by volume. Stiffness and compressive strength decrease with increasing porosity; values of the Young’s modulus range are approximately 1.5–2.3 GPa (60% porosity), 0.8–1.1 GPa (70% porosity), and 0.35 GPa (80% porosity). The yield strength is higher than trabecular bone and suitable for prosthetic applications. The effect of the particle size and compaction pressure on the scaffold thickness is less marked at high porosity values (negligible for 80% porosity).

In the study by Luo et al. [47], porous tantalum scaffolds with different pore sizes (100–200, 200–400, 400–600, and 600–800 μm) and different porosities (25%, 55%, 75%, and 85%) were produced by means of computer-aided design and 3D printing techniques; they were investigated using in vitro and in vivo studies. Ta scaffolds with of 400–600 μm pore size showed higher ability to promote cell adhesion, proliferation, and osteogenic differentiation in vitro; moreover, these scaffolds demonstrated better performances in vivo as to bone ingrowth and device integration. Through computational fluid dynamics analysis, it was possible to establish that a 400–600 μm pore size allows suitable permeability and surface area to improve cell adhesion and proliferation, resulting in enhanced osteogenesis and osseointegration.

#### 3.1.3. Additive Manufacturing

Medical therapies are moving toward an autologous (personalized) rather than heterologous approach; thus, the need emerges to find manufacturing techniques that easily allow the customization of implantable devices with respect to the specific anatomical characteristics of each patient. In this context, additive manufacturing (AM) appears as a versatile and effective technology to fabricate porous scaffolds intended for tailored orthopedic applications.

Additive manufacturing is the process of joining/connecting materials through successive stratification to obtain objects starting from digitalized 3D models [48]. In particular, AM allows obtaining highly customized porous structures with complicated geometries, which accurately correspond to the desired anatomical shape. Furthermore, scaffold porosity can be easily tailored to meet the compressive strength and elastic modulus of bone tissue, avoiding the stress-shielding phenomenon [16]. 

The ASTM classifies AM techniques into seven groups: vat photopolymerization, material jetting, binder jetting, material extrusion, sheet lamination, direct energy deposition, and powder bed fusion. These classes differ depending on the materials and the process used for layer deposition [49,50].

Basically, the AM technique requires three main steps [47]: the creation of the 3D model of the object to reproduce, sending the file to the printer, and realization of the physical object layer by layer.

Over the years, many AM techniques have been developed, including selective laser melting (SLM), electron beam melting (EBM), direct metal deposition (DMD), direct metal printing (DMP), fused deposition modeling (FDM), direct metal writing (DMW), and binder jetting (BJ). In particular, SLM and EBM are the most widely used AM techniques for the fabrication of metallic porous scaffolds due to their high precision, efficacy, and good stability [51,52]. In these two techniques, both included in the powder bed fusion category, metal powders are sintered/melted by a different energy source, i.e., an electron beam for EBM and a laser beam for SLM. These systems consist of two platforms, a material delivery platform and a build platform, both enclosed into a chamber. The first platform constantly supplies new material, while a blade gradually removes its excess; the intended object grows onto the build platform by melting metallic powders layer by layer. Once a layer is formed, the platform descends, and a new layer of metal powder is added by the delivery platform. The final step consists of cutting the object formed from the support after obtaining the whole structure.

### 3.2. Unit Cells

In order to promote bone tissue regeneration, porous scaffolds have to be able to integrate with the patient’s body and promote tissue growth. It is worth noting that both the geometry and the mechanical properties of the trabecular prosthetic implant influence the response of bone tissue. In other words, scaffold performances can be optimized by controlling pore size and shape depending on the manufacturing technique. For example, CVD allows managing only the porosity and the pore average size, but it does not assure specific geometrical characteristics such as the shape of the pores and their interconnection. Similar limitations are also associated with PM, while 3D technologies, such as AM ones, allow directing geometric characteristics with precision and accuracy, which both depend on the resolution of the device [53].

In designing scaffold porosity, it is important to distinguish the presence of open or closed cells. In the closed cell scaffolds, each cell is surrounded by a thin wall; in the open cell structures, cells are connected with each other, allowing bone tissue infiltration. The first structures are the result of a random formation process, in which the size, shape, and location of the pores are variable. Therefore, the resulting scaffold exhibits limited porosity and substantial inhomogeneity in pore size and shape. 

With regard to porous systems, there are three types of structures: (i) partially or fully coated porous solid substrates; (ii) fully porous materials; (iii) porous metal segments joined to a solid metal core. There are multiple applications for both substrates and fully porous structures such as spinal fixation devices, fracture plates, screws, craniofacial implants, maxillofacial implants and bone grafts. Implants consisting of a solid core and structures with a porous coating are more appropriate when the porous metal does not provide sufficient mechanical strength to bear physiological loads, as in the case of dental implants or joint prostheses [54].

To design appropriate scaffolds for bone tissue, computational mechanobiological models have been developed as an alternative to the traditional experimental approach. Bone cells are sensitive to mechanical loads; thus, they regulate some functions (i.e., proliferation, differentiation, synthesis, and remodeling of the extracellular matrix).

Several models have been proposed to determine the best geometry for a porous scaffold. In the study published by Rodríguez-Montañoa et al. [53], the optimal geometry was based on four different unit cells (truncated cuboctahedron, truncated cube, rhombic dodecahedron, and diamond). Scaffolds of different geometries were compared under seven loading conditions, measuring the amount (percent) of the volume occupied by bone ingrowth. The four geometries were defined using finite element (FE) analysis. Two fundamental parameters were fixed: the unit cell dimension Q (637 μm) and the length (μm) of the beams for each cell (L1 = 166.39, L2 = 263.85, L3 = 356.09, and L4 = 275.83). The amount of bone ingrowth increased while the load also increased; the truncated cube exhibited the worst results (less than 20% at 1.5 MPa), while the other cells gave similar performances (40–50% at 1.5 MPa). Indeed, this numerical approach dramatically simplified the real condition of bone–material interactions, for which many stimuli other than mechanical ones can affect bone response (i.e., angiogenesis, oxygen and nutrient supply, and biochemical signals).

## 4. Medical Applications of Trabecular Tantalum

**Figure 2:**
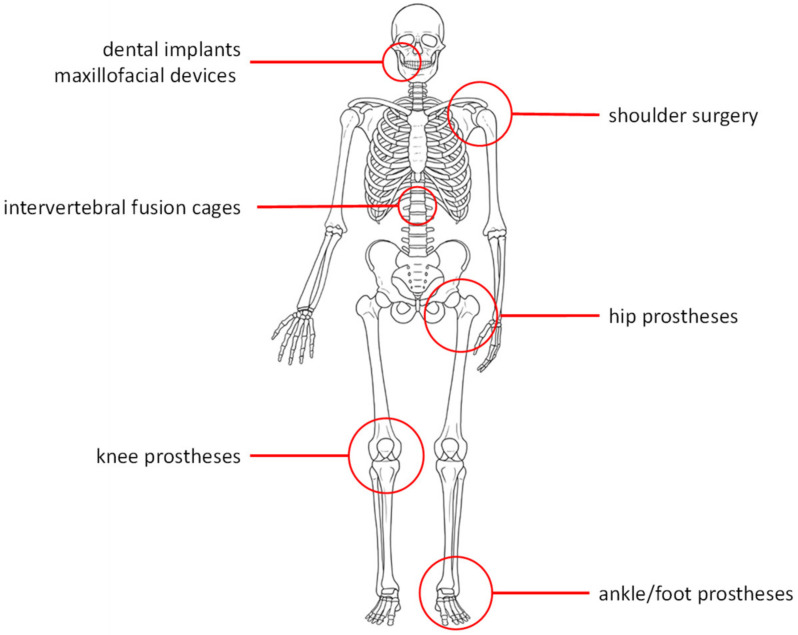
Tantalum devices for endosseous applications.

Over the last decades, porous Ta implants have been widely exploited for several endosseous applications (Figure 2). The properties of such implants (elastic modulus similar to cancellous bone, suitable mechanical strength, excellent corrosion resistance, and osseointegration) assured promising performances in the field of orthopedic and dental applications. In particular, orthopedic applications include hip and knee prostheses, spinal fusion devices, shoulder reconstruction, and foot and ankle surgery [54,55].

### 4.1. Hip Restoration and Total Hip Arthroplasty (THA)

Trabecular Ta components are used to partially or totally replace the hip joint. Generally, there are three different kinds of prosthetic devices: porous Ta rods, uncemented porous Ta monoblock acetabular cups, and porous Ta augments. These devices are characterized by low elastic modulus, high surface friction, and remarkable osseointegration properties.

The insertion of Ta rods represents an effective treatment for early-stage osteonecrosis of the femoral head. As expected, they completely integrate with the host bone, by providing structural support for the necrotic regions. These devices aim at preserving the femoral head and prevent worsening to procrastinate the total hip replacement [16]. The study published by Liu et al. [56] investigates the use of this device in 149 patients affected by osteonecrosis. After 3 years, the follow-up revealed excellent clinical results. The device was correctly functioning in approximately 63.1–68.8% of cases; for the other patients, the femoral head continued to collapse even after porous Ta rod insertion, thus resulting in implant failure. Overall outcomes markedly depend on the preoperative conditions, i.e., location and size of the bone lesion.

A finite element analysis of the femoral head, simulating lesions of different diameter (15, 20, and 30 mm) treated with Ta rods, was proposed in [57]. The surface of the femoral head was designed with 3 mm of cortical bone thickness and spongy bone inside. The elastic modulus for the bone and the prosthesis were 15,000 MPa and 3000 MPa, respectively. Three femoral head models were designed to represent normal condition, necrotic condition, and bone repair condition. The stress distribution within the femoral head was assessed across four layers (from bone surface to subchondral bone) depending on the presence or absence of lesions, their size, and the contribution of the insertion of porous Ta rods. The peak load (91.3 MPa) was reached in the model with the largest lesion (30 mm). Interestingly, after Ta rod insertion, the three models affected by osteonecrosis showed a stress pattern similar to that of the nonpathological model. The presence of the device allowed reducing the mean stress in all pathological models: from 34.4 ± 8.7 MPa to 24.4 ± 9.6 MPa for the smallest lesion (15 mm); from 33.9 ± 8.5 MPa to 26.8 ± 8.2 MPa for the mean lesion (20 mm); from 38.4 ± 9.9 MPa to 26.5 ± 8.9 MPa for the largest lesion (30 mm).

Since porous Ta structures have a low failure rate in diseases leading to severe bone loss, they can be also used for the treatment of periacetabular lesions due to neoplastic processes [58].

By means of MRI and CT imaging, it is possible to check the location of the lesion and to measure its size to select the most appropriate surgical treatment, e.g., Ta rods insertion or THA.

Uncemented porous Ta monoblock acetabular cups are an effective alternative to the conventional cemented polyethylene acetabular cups in the context of THA and congenital hip dislocation. The effectiveness of porous Ta monoblock acetabular components was assessed in 82 patients, who underwent THA, through a follow-up of 7.3 years [59]. In postoperative radiographic images, complete implant contact between the prosthesis and cortical bone was observed in 54 patients; conversely, the presence of a gap (0.2–5 mm) was noticed in the remaining patients. After 24 weeks, X-ray imaging revealed the absence of empty space in all patients. No patient experienced dislocations, presence of debris or related complications; all patients were able to resume their normal activities, and no area of osteolysis was observed in any implant at the last follow-up.

The release of metallic and polymeric debris is generally associated with the acetabular components due to the contact between the internal polyethylene module and the external metallic surface. Debris can migrate toward the periprosthetic region and be the cause of osteolysis and aseptic loosening of the implant. Other studies confirmed excellent performance of these implants in terms of absence of osteolysis aseptic loosening and stability [60,61,62,63].

A recent systematic review and meta-analysis compared the use of trabecular metal and traditional metal cups for acetabular revision surgery. The comparison was based on device survival and incidence of adverse events [64]. A total number of 13,864 THA revisions were included in the meta-analysis: 5619 with TM cups and 8245 with traditional metal. Despite the hypothesis preliminarily formulated by the authors (“TM cups have better survival rates than non-TM cups in acetabular revision surgery”), they did not find significant differences in cup survival between TM or non-TM cups when using re-revision for any reason or aseptic loosening as endpoint. The overall incidence of adverse events accounted for 6.8% for TM cups and 9.0% for non-TM cups; the incidences of aseptic loosening and infection were lower for TM cups; the incidence of dislocation was lower for non-TM cups. In light of these findings, the authors recommended great caution in the selection of the appropriate material for THA revision.

### 4.2. Knee Surgery

Trabecular Ta can be used for the production of the devices for total knee arthroplasty (TKA). There are three different types of prostheses for the knee: uncemented porous Ta monoblock tibial components, porous Ta metaphyseal cones, and porous Ta patellar components.

In TKA, cementless monoblock tibial components are commonly used despite their limitations, i.e., high cost, complex surgery, and unsatisfactory performance in terms of osseointegration. The use of porous Ta cementless monoblock components showed much better performance than traditional ones, with favorable clinical outcomes over both short and long term [16]. The authors of [65] investigated the effectiveness of this kind of devices in a group of 95 patients, with an average follow-up of 4.5 years. In particular, 91 patients suffered from knee osteoarthritis, one patient suffered from hemophilia, and another one suffered from rheumatoid arthritis. All patients received both tibial and femoral components. Compared to the preoperative condition, the patients experienced a greater possibility of knee flexion: an average value of 127° to 138°. Moreover, tibial components maintained their integrity and location, and expressed excellent integration with the surrounding bone. It was not necessary to fix the device with screws to ensure stability and prevent micro-movements.

Porous Ta cementless tibial components and polymethylmethacrylate (PMMA) cemented tibial components were compared in two different groups of patients [66]. The study aimed at identifying which of the two kinds of prosthetic implants was more efficient in terms of knee functionality, by evaluating the following indicators: KSS index (Knee Society Scores) and WOMAC osteoarthritis index (Western Ontario and McMaster University). The use of cementless porous Ta monoblock tibial components was associated with a slightly higher functional score, fewer radiolucent lines, and shorter operation [67]. Another study [68] analyzed the clinical and functional outcomes in 33 patients (average follow up of 11.5 years), who underwent TKA with cementless tibial components made of porous Ta. Among all patients, 31 were suffering from osteoarthritis and two were suffering from post-traumatic arthritis. In all patients, radiographic images did not detect any adverse effect due to osteolysis, aseptic loosening, or collapse of the prosthesis. Furthermore, the average KSS index increased from 56 to 93, confirming the long-term effectiveness of the prosthetic device.

In the paper published by Kamath and coworkers [69], Ta tibial metaphyseal cones were applied to 63 patients to treat massive tibial bone loss. The mean KSS improved significantly from 55 preoperatively to 80 points at the time of the latest follow-up, with durable clinical outcomes and radiographic fixation. The authors concluded that the bone tissue ingrowth of these devices offers the potential for successful long-term structural support in complex knee reconstruction.

In another paper [70], porous Ta components were used to fix patellar bone defects in 23 patients with a mean follow-up of 7.7 years. In all cases, prosthetic devices showed good osseointegration, a good KSS value (82.7), and absence of aseptic mobilization. The success (or failure) of such an implant strongly depends on the amount of bone in contact with it: the greater this quantity, the lower the failure rate of the prosthesis.

### 4.3. Spinal Surgery

Porous Ta scaffolds were used in spinal applications for both cervical and lumbar interbody fusion. In [71], the clinical results from 99 patients, who underwent cervical discectomy and fusion with the implantation of porous Ta cages, were illustrated. For all patients, the implant was found in the right position even at the long-term follow-up and correctly functioning. It was concluded that porous Ta cages assured long-term clinical benefits and a very low rate of complications.

The aim of the study in [72] was to evaluate the clinical outcomes of posterior lumbar interbody fusion (PLIF) procedure using a porous Ta implant. A group of 52 subjects were treated without the help of bone grafting. All patients reported a major physical and functional benefit 1 year after surgery, thanks to excellent osseointegration and stability.

The application of porous tantalum in spinal surgery was reviewed by Hanc et al. [73]; these authors stated that trabecular metal is effective for achieving anterior and posterior interbody lumbar fusion, with good clinical outcomes. Vice versa, unsatisfactory results were achieved for cervical interbody fusion; the study published by Kasliwal and coworkers [74] demonstrated that a standalone porous tantalum device is not the ideal approach because of the low rate of arthrodesis and the risk of failure.

### 4.4. Shoulder Surgery

Applications of trabecular Ta include shoulder prostheses and monoblock glenoid components. A group of 51 patients with proximal humerus fracture underwent total shoulder arthroplasty (TSA) [75]. After a mean follow-up of 3 years, a healing rate of 92% was found without prosthetic loosening phenomena and infections. Furthermore, this device allowed good mobility recovery. More recently, Sasanuma et al. [76] compared the clinical results of the use of nonporous and porous Ta prostheses for treating humerus proximal fractures in 41 elderly subjects. Porous devices evidenced much better performances, allowed greater ranges of motion for the shoulder, and exhibited higher osseointegration level.

The study by Merolla et al. [77] compared the first and the second (trabecular Ta) generation of glenoid components for TSA in a group 40 consecutive patients with a mean follow-up of 3 years. A noticeable difference before and after surgery was observed using the second-generation components: the average constant score (CS) increased from 23.2 to 69.8, and the American Shoulder and Elbow Surgeons (ASES) score increased from 24.1 to 93.4. Most of the subjects (77.5%) returned to their lifestyle, work included. For the second-generation components, adverse phenomena were not noted with regard to the collapse of the prosthesis, debris, and incorrect placement.

### 4.5. Foot and Ankle Surgery

Porous Ta scaffolds were exploited for foot and ankle surgery with some clinical success starting from the beginning of 2020s [78,79]. The paper published by Tiusanen et al. [80] described the clinical outcomes in a group of 104 patients, who underwent total ankle arthroplasty (TAA). The effectiveness of porous implant was assessed over a 5 year follow-up. A very low rate of osteolysis and loosening of components was detected, but a non-negligible number of complications were registered (nearly 20%). Surely, this kind of prosthesis needs further investigations from both morphological and functional points of view to be validated as an alternative to traditional bone grafts for foot and ankle surgery.

### 4.6. Dental Implants

In dental applications, trabecular Ta is used for coating implants made of different materials (e.g., Ti). This new kind of dental implant can improve clinical outcomes. Generally, implant surfaces are produced with adequate roughness to get large bone-to-implant contact and stable fixation, thus reducing peri-implant bone loss. In addition to traditional manufacturing techniques (e.g., sandblasting, acid etching, plasma spraying, or combinations thereof [81]), porous Ta can be used to cover a different metallic core (i.e., titanium alloy Ti6Al4V). The porous coating is typically made of ~2% vitreous carbon scaffold and ~98% Ta; it is then applied over the central titanium core. Thanks to its regular porosity, the Ta coating allows for rapid budding and endothelial cell growth in response to the gradient of angiogenic and anabolic growth factors within the scaffold. The size of the open-pore structure was designed to accommodate fast neovascularization, which is critical to allow the recruitment of osteoblast precursors and their differentiation into osteoblastic cells; these cells then grow and secrete bone matrix [82]. New bone formation was improved, resulting in immediate and early loading of the implant [21]. 

This kind of implants was clinically evaluated in the study [83]; out of 37 devices inserted, only one failed the osseointegration (2.7%). This was a preliminary investigation evidently limited by the number of subjects and the duration of the follow-up; nevertheless, it represents an excellent basis for further assessing porous Ta-based implants in terms of osseointegration potential, bone growth, and biocompatibility.

The right design of any surface in direct contact with bone tissue is of paramount importance in order to promote osseointegration and angiogenesis [56]. Indeed, osseointegration potential has to be considered as a fundamental requirement for the success of dental implants. The retrospective study by Edelmann et al. [84] compared the results obtained from the insertion of Ti alloy dental implants coated and not coated with porous Ta. A total number of 205 implants in 82 patients was considered; no failure was found in the group of Ta-coated devices, while three implants failed in the other group. The authors stated that Ta-coated implants showed less peri-implant bone loss compared to traditional devices.

Another interesting paper was recently published [85] to evaluate the stress distribution due to the presence of a trabecular Ta implant and a Ti implant in the mandibular bone using 3D finite element (FE) analysis. Generally speaking, the design characteristics of any implant affects stress distribution over the bone tissue, and this can be a decisive factor for success or failure. In this study, a porous Ta implant and Ti solid implant were compared under different simulated loading conditions: 100 N vertical loads on the left first molar (VM), 100 N vertical loads on the lower incisors, and 100 N loads inclined (45°) and applied to the center of the left first molar (IM). The FE results established that the loading site was the most important parameter influencing stress distribution. Furthermore, inclined loading on the molar teeth induced higher stress levels on the cortical bone around the implant closest to the loading site in both models, while vertical loads on the first molar tooth produced the lowest stress. Moreover, FE analysis revealed that the trabecular Ta implant reduced the rate of deformation around the cortical and trabecular bones. Therefore, trabecular implants were able to improve the clinical success by reducing the marginal bone loss.

With regard to the application of porous Ta in the dental filed, the paper published by Fraser et al. [86] has to be mentioned, which partly reduced the expectations over the exceptional results previously obtained in vivo (rabbit). The authors did not find significant differences due to the presence of porous Ta when measuring the impact of implant type, bone region, and time on implant stability quotient (ISQ), hardness, and elastic modulus of newly grown bone. Indeed, these conclusions were followed by similar results obtained in the simulation study by Magic et al. [87]. Even though the tone of the conclusions stated by the authors (“TMTM implant can achieve good primary implant stability in terms of insertion torque and resonance frequency analysis”), their findings demonstrated that the presence of porous Ta did not improve implants performances in terms of stability (ISQ) and insertion torque. On the contrary, Bencharit and coworkers demonstrated that porous Ta-based implants resulted in a significantly higher expressions of genes specific to neovascularization, wound healing, and osteogenesis than Ti-based implants [88]. The authors eventually declared that the better performances of porous Ta over the healing phase could be due to its capacity to stimulate a more favorable gene expression profile. The same group of authors published an interesting paper [89] to evaluate the expression patterns of a panel of genes associated with osteogenesis and wound healing in osteopenic patients. Patients received titanium or porous tantalum cylinders, and the pathway of gene activation was checked at the beginning of osseointegration. Tantalum was able to induce an earlier osteogenic genes activation. This result allowed hypothesizing a reduction in the risk associated with the application of dental implants in osteopenic patients.

## 5. Bone Tissue Regeneration Induced by Tantalum

In the context of bone tissue regeneration, different materials can be used for manufacturing prosthetic devices: ceramics, polymers, and metals. Ceramics and polymers show promising bioactivity characteristics; however, the low mechanical strength of polymers and the brittleness of ceramics represent major limitations. Currently, metallic scaffolds are the most adequate for load-bearing devices; various porous structures and coating surfaces are made of titanium and its alloys, thus improving the osseointegration potential. Although these materials exhibit good clinical outcomes, they are affected by some weaknesses: possible release of metal ions caused by corrosion, low osteoconductivity, low friction coefficient, high elastic modulus, and low porosity. A metal implant that fails to promote sufficiently strong bonds with the bone tissue leads to loosening even in the absence of infection (aseptic loosening). Porous Ta seems to be able to overcome these limitations [11,47].

A number of in vitro and in vivo investigations have been published to demonstrate that porous Ta is not only a biocompatible material, but it can also assure good osteogenic and osteoconductive potential. For example, the paper by Guo et al. [90] evaluated the osteogenesis and osseointegration of Ta scaffolds manufactured by SLM; cytocompatibility assays were performed in vitro with human bone mesenchymal stem cells (hBMSCs), and the osseointegration ability was assessed in vivo in the animal model (New Zealand rabbit). Porous Ta scaffolds showed cell adhesion and proliferation higher than the control group (e.g., porous Ti6Al4V); moreover, the osteogenic differentiation of hBMSCs was enhanced in the Ta group. In vivo, new bone formation was higher for Ta scaffolds than Ti6Al4V ones, with increased bone ingrowth and osseointegration. Similar results were reported elsewhere [91,92,93].

The role of trabecular tantalum in regulating the behavior of BMSCs to enhance bone regeneration was recently deepened in the work by Zhou and Liu [94]. This was an exhaustive review elucidating the beneficial features of porous tantalum from a general point of view, even describing its superior ability to favor osteogenic differentiation through the regulation of specific genes and the activation of signaling pathways. Therefore, the authors supported the use of porous tantalum for bone regeneration and tissue repair after injury.

The study in [95] examined the effects of porous Ta on surrounding periprosthetic remodeling around the femoral stem in a group of 118 patients; they randomly received a cementless Ti6Al4V femoral stem for metaphyseal fixation or a conventional titanium stem with fiber mesh coating. Following hip replacement surgery, the periprosthetic bone mineral density (BMD) was monitored by densitometry 1 week after surgery, and 6, 12, and 24 months later. The relative change in BMD was calculated in each of the seven Gruen zones [96]. At each planned follow-up, a significant difference in relative change of BMD values between the two types of systems was detected; trabecular Ta reported a smaller decrease in BMD. Apart from a postoperative infection, no other complication was found, and it was not necessary to resort to revision surgeries. Moreover, no evidence of osteolytic lesions around the stems or prosthetic loosening was reported. In this study, trabecular Ta was found to be markedly superior with respect to the conventional titanium stem in terms of bone remodeling.

A retrospective study [97] compared the outcomes of hip prosthesis revision using two different acetabular cups: Ti6Al4V alloy coated with hydroxyapatite (HA) and porous Ta. Out of 286 patients, 207 (214 prostheses) received the Ti alloy acetabular implant, and 79 (81 prostheses) had the trabecular Ta one. Minimum follow-up was 24 months, with an average of 51.8 months for the first group and 35.4 months for the second group. Even though the failure rate was similar in both groups (8% for Ti6Al4V and 6% for porous Ta), a difference in terms of bone growth was noticed; for Ti alloy devices, bone growth occurred only in the periprosthetic area, while trabecular Ta allowed bone ingrowth within the porous structure. This study confirmed that porous Ta is a suitable material to be used for the production of both acetabular components and the stem for total hip prosthetic devices. 

Another study [98] compared uncemented monobloc acetabular cups in porous Ta (TM) and porous-coated Ti in 86 patients over an average follow-up of 12 years. In particular, the porous acetabular cup was obtained by compressing the inner polymeric component (ultrahigh-molecular-weight polyethylene, UHMWPE) directly against the outer porous Ta shell. Surface porosity was 75–80% with an average pore size of 550 μm. Ti acetabular cups were similarly obtained, but with a coating surface made of three layers of Ti particles (200–300 μm diameter), with a porosity of 30–50%. Twelve years after surgery, no implants migrated in both groups; two TM patients (4%) and 13 Ti patients (33%) revealed radiolucency around the cup; one cup (2%) was revised for aseptic loosening in the control group (Ti). Therefore, once again, it was possible to ascertain that porous Ta prostheses exhibit excellent osseointegration properties and survival rate (100% after 12 years).

To assess the osteogenic properties of porous Ta in vitro and in vivo, Ta-implanted entangled porous titanium (EPT) surfaces, produced by plasma immersion ion implantation and deposition, were compared to Ca-implanted and unimplanted EPTs [99]. No significant difference among the three materials was observed with regard to the yield strength and elastic modulus, and the surface topography. On the other hand, Ca- and Ta-implanted groups enhanced the promotion of MG-63 cell (from human osteosarcoma) viability, proliferation, differentiation, and mineralization more than the unimplanted surfaces. A higher level of osseointegration of both Ca- and Ta-implanted EPT devices was ascertained in vivo (rabbit as animal model) by micro-CT evaluation, pushout test, sequential fluorescent labeling, and histological analyses. However, the Ta-implanted group showed a more stable and continuous osteogenic activity. The authors concluded that Ta-implanted EPT is a highly efficient graft material for bone regeneration.

The osteogenic potential of titanium implants, coated and noncoated with porous tantalum, was also assessed in a gap-healing model in the rabbit tibia [100].

A recent study comparing the performances of porous Ta with those of pure Ti was published by Piglionico et al. [101]. Mesenchymal stem cells from the dental pulp (DPSC) were incubated on Ta, and on smooth and rough Ti; cell adhesion, proliferation, osteodifferentiation, and mineralized matrix production (after 3 weeks) were assessed. The 3D porous Ta surface demonstrated a much higher capacity of improving cell functions than Ti substrates, and this result confirmed the enhanced osteogenic capacity provided by Ta. 

In the paper by Zhang et al. [102], hierarchical tantalum scaffolds were produced to mimic the structure of natural bone enhancing osseointegration. After anodization to get nanotubes, porous tantalum scaffolds demonstrated improved hydrophilicity and protein absorption capacity. Moreover, MC3T3-E1 preosteoblastic cells were cultured onto these scaffolds, showing an upregulation of osteogenic marker gene (Osterix, Runx2, COL-I) after 7 days. Following implantation into the femurs of New Zealand rabbits, the authors found a superior early osseointegration.

## 6. Discussion

As documented in the previous sections, porous tantalum can represent an excellent choice for the production of endosseous implantable devices, with specific regard to orthopedic and dental fields. The regular porous structure might be able to minimize the stress-shielding effect, as well as promote bone ingrowth for optimal and durable fixation. Thanks to the customizable design, surgical times can be reduced. Moreover, porous tantalum exhibits good biocompatibility associated with high corrosion resistance and bioactivity. As proven both in vitro and in vivo [103], intrinsic bioactivity can be further improved by surface modifications, which can favor cell adhesion, proliferation, and differentiation, eventually improving new bone growth. An exhaustive review on tantalum surface modifications was recently published by Wang et al. [104]. Another interesting review paper addressed the physicochemical, mechanical, and biological features of porous tantalum for better performances in oral implantology [105]. Moreover, it is also possible to improve the antiadhesive and antibacterial properties [18,106,107,108]. In addition, the porous structure can be an exceptional vehicle for drug delivery systems [109]. 

On the one hand, the ability of trabecular tantalum to establish beneficial interactions with cells and tissues has been thoroughly investigated, and its potential role in favoring bone regeneration has been ascertained. Many papers discussed a number of possible applications in wound healing and tissue regeneration; in addition to those mentioned above, we report the work by Zhao et al. [110], who exploited the angiogenic potential of a scaffold obtained by combining porous tantalum and gelatin nanoparticles. Another interesting application was recently proposed by Liu et al. [111]; they enriched the surface of a porous polymeric/ceramic/polydopamine scaffold with tantalum to get a bioactive material suitable for repairing bone defects thanks to its osteogenic potential.

On the other hand, the clinical exploitation of porous tantalum is still far from achieving unequivocal outcomes. As previously discussed, the work by Shen et al. [65] did not reveal a clear superiority of the acetabular cups obtained with porous tantalum compared to the traditional ones. Indeed, other authors claimed successful applications of porous tantalum. Nevertheless, it has to be stated that any valuable comparison of clinical results is hampered by the number of different applications using different devices, the variety of patients, and the differences in follow-up duration.

The review paper published by Han and coworkers [5] clearly depicted advantages and disadvantages of both tantalum and titanium for real surgical purposes, considering manufacturing techniques and costs. It can be concluded that porous metals surely possess favorable features for the production of endosseous implantable devices; actually, the practical experience with titanium is much more consolidated than that with tantalum.

## 7. Conclusions

Despite all well-acknowledged beneficial features that characterize trabecular tantalum, it is still suffering from the following limitations:

− lack of regulations on the use of the devices produced by AM technique;
− more resources used in prosthesis customization than in describing the clinical treatment;
− lack of long-term clinical outcomes.

Moreover, the application of customized implants needs intensive cooperation between medical doctors and (biomedical) engineers, which can be sometimes difficult to manage. Nevertheless, no clinical translation will be possible in the future without the effective integration of life sciences, medicine, and engineering to address major challenges in medicine and healthcare.

## Figures and Tables

**Table 1:** Main mechanical properties of tantalum and other metals for biomedical use [22].

Metal	Elastic Modulus (GPa)	Yield Strength (MPa)	Tensile Strength (MPa)
Tantalum	185	138	207
cp Titanium	110	485	760
316 L stainless steel	190	331	586
Co-Cr alloy	210	448–648	951–1220

**Table 2:** Atomic number and density of some metals [22].

Metal	Atomic Number	Density (g/cm^3^)
Tantalum	73	16.6
Titanium	22	4.5
Iron	26	7.9
Cobalt	27	8.9

**Table 3:** Mechanical properties of trabecular Ta [11,22].

Parameter	Value
Elastic modulus	2.5–3.9 GPa
Ultimate strength	50–110 MPa
Yield strength	35–51 MPa
Compressive strength	50–70 MPa
Tensile strength	63 MPa
Bending strength	110 MPa

## Data Availability

Data sharing not applicable.
